# ^99m^Tc-3P-RGD2 molecular imaging targeting integrin *α*_v_*β*_3_ in head and neck squamous cancer xenograft

**DOI:** 10.1007/s10967-015-3928-5

**Published:** 2015-02-14

**Authors:** Bing Yan, Fan Qiu, Ling Ren, Haojie Dai, Wei Fang, Haibo Zhu, Feng Wang

**Affiliations:** 1 Department of Nuclear Medicine, Beijing Tongren Hospital, Capital Medical University, Beijing, 100730 China; 2 Department of Nuclear Medicine, Nanjing First Hospital, Nanjing Medical University, 68 Changle Road, Nanjing, 210006 China; 3 Cardiovascular Institute & Fu Wai Hospital, Peking Union Medical College & Chinese Academy of Medical Sciences, Beijing, 100037 China; 4 State Key Laboratory of Bioactive Substance and Function of Natural Medicines, Institute of Materia Medica, Peking Union Medical College & Chinese Academy of Medical Sciences, Beijing, 100050 China

**Keywords:** Head and neck squamous cell carcinoma, Integrin *α*_v_*β*_3_, Molecular imaging, ^99m^Tc-3P-RGD2, Angiogenesis

## Abstract

^99m^Tc-3P-RGD2 and SPECT/CT were valuable tools for selecting patient likely benefit from integrin *α*_v_ *β*_3_ blocking therapy. To evaluate the feasibility of ^99m^Tc-3P-RGD2 imaging to detect head and neck squamous cell carcinoma, ^99m^Tc-3P-RGD2 was prepared and the relationship between its accumulation and integrin *α*_v_ *β*_3_ expression in nude mice bearing HEP-2 or CNE-1 carcinoma xenograft were analyzed. This study demonstrated that ^99m^Tc-3P-RGD2, with high affinity to integrin *α*_v_ *β*_3_, will provide basis for *α*_v_ *β*_3_ involved individual therapy.

## Introduction

Head and neck squamous cell carcinoma (HNSCC) was reported as the fifth most common cancer worldwide with high morbidity and low survival [1]. In 2012, about 7,000,000 people have been diagnosed and more than 370,000 died of head and neck cancer, treatment for head and neck squamous cancer is a great challenge worldwide [2]. During 5-year follow-up, the recurrence of patients with advanced cancer is 20–25 % [3, 4]. Second primary cancers occur in 3–5 % of cases per year [5]. Early diagnosis and proper treatment is not only to decrease the morbidity but improve the life quality of patients.

Several imaging were performed for detecting HNSCC, including CT scan, CT perfusion, MRI, MRI perfusion, and ^18^F-FDG PET/CT. Most HNSCC detected by CT scans were at the advanced stage. MRI has its own limitations, like long scan time, fear of confined spaces. CT perfusion and MRI perfusion could not be use wildly because of the high cost of money. Although ^18^F-FDG PET/CT is the most commonly used for oncologic purposes, ^18^F-FDG uptake reflects glucose metabolism and can be observed in several normal tissues with wide variability of the normal pattern influence its power. New effective method is needed.

It is reported that angiogenesis is critical in the development of HNSCC. Several studies have suggested that anti-angiogenesis is important in the prognosis in HNSCC [6–8]. Integrin *α*_v_ *β*_3_, which moderates tumor angiogenesis, specifically binding to arginine–glycine–aspartic acid (RGD) makes RGD a promising tracer to detect tumor and monitor patients receiving anti-angiogenic drugs or *α*_v_ *β*_3_ antagonists [9]. Recently, encouraging results have been achieved with integrin *α*_v_ *β*_3_ antagonists in various malignant tumors [10–12]. The use of etaracizumab [13–15], which is anti-angiogenesis therapy, has been evaluated in clinical trials. A variety of radiolabeled RGD for single photon emission computed tomography (SPECT) and positron emission tomography (PET) have been developed [16–21]. Nowadays a new radiolabeled tracer, [^99m^Tc(HYNIC-3P-RGD2)(tricine)(TPPTS)] (^99m^Tc-3P-RGD2: 6-hydrazinonicotinyl; 3P-RGD2   =   PEG4-E[PEG4-c(RGDfK)]2; PEG4   =   15-amino-4,7,10,13-tetraoxapentadecanoic acid; and TPPTS   =   trisodium triphenylphosphine-3,3′,3″-trisulfonate), was used in a variety of tumors [22–26]. This study was to evaluate the feasibility of ^99m^Tc-3P-RGD2 detecting HNSCC in nude mice tumor model and the possibility of choosing proper individual therapy.

## Materials and methods

### Radiolabeling and quality control

Cyclic RGD peptide 3P-RGD2 was obtained from the School of Health Sciences, Purdue University, USA. Na^99m^TcO_4_ was purchased from the Beijing Atom High Tech Co.,Ltd. To a lyophilized vial containing 20 μg of 3P-RGD2 was added 1.0 mL of Na^99m^TcO_4_ solution (370–1111 MBq/mL). The vial was heated at 100 °C for 20 min in a lead-shielded water bath. After radio-labeling, the vial was kept at room temperature for 10 min. A sample of the resulting solution was tested by radio-high performance liquid chromatography (HPLC, HP Hewlett Packard Series 1100, USA) at the Peking University Medical Isotopes Research Center. The radiochemical purity was >95 % for ^99m^Tc -3P-RGD2.

### Stability in vitro

The stability of ^99m^Tc-3P-RGD_2_ in newborn calf serum was determined after incubating the radiolabeled compound (37 MBq) in 2 mL human serum at 37 °C. Every 20 μL mixture was injected directly into the radio-HPLC to analyze the radiochemistry purity, which was followed by radiolabeling efficiency analysis at 0 min, 3, 4, and 6 h.

### Cell culture

Human laryngeal cancer cells HEP-2 and human nasopharyngeal cancer cells CNE-1 (Cancer Hospital, Chinese Academy of Medical Sciences, China) were maintained at 37 °C and 5 % CO2 in RPMI 1,640 medium containing 10 % fetalbovine serum (FBS).

### Western blot

The cells were lysed with cell lysis buffer (150 mmol/L NaCl, 1 % [vol./vol.] Triton X-100, 0.5 % [wt/vol.] sodium deoxycholate, 0.1 % [wt/vol.] SDS, 50 mmol/L Tris–HCl, pH 7.4) containing protease inhibitor cocktail (Sigma). The lysate was subjected to SDS-PAGE, transferred to poly (vinylidene fluoride) (PVDF) membranes, and incubated with the primary antibodies [rabbit anti human integrin beta3 (Chemicon, Temecula, CA, USA) and mouse anti human beta-actin (Chemicon, Temecula, CA, USA)], followed by horseradish peroxidase-conjugated secondary antibody (Amersham, Little Chalfont, Bucks, UK). The bound antibody was visualised using enhanced chemiluminescence reagents (Pierce, Rockford, USA). The integrated density of each lane was quantified by Image J (Image Processing and Analysis in Java) program.

### Animal model

Female BALB/c nude mice (4–5 weeks of age, 15–20 g of weight) were purchased from the Department of Animal Experiment, Chinese Academy of Medical Sciences. The mice were subcutaneously implanted with 3 × 10^6^ the HEP-2 cells (6 mice) or 2 × 10^5^ the CNE-1 cells (6 mice) in 0.1 mL of saline into the right upper shoulder flanks. All procedures were performed in a laminar flow cabinet using the aseptic technique. Fifteen to nineteen days after inoculation, the tumor size was 1–1.5 cm, the tumor-bearing mice were used for biodistribution and imaging studies. Capital Medical University Animal Care and Use Committee approved the animal experiments.

### Biodistribution

The biodistribution of ^99m^Tc-3P-RGD2 in HEP-2 tumor bearing mice and CNE-1 tumor bearing mice was evaluated in groups of 3 mice per time point at 60 min and 120 min after injection of approximately 0.55–0.74 MBq of ^99m^Tc-3P-RGD2 in 0.1 mL saline via tail vein. From all mice, tumors and tissue samples (blood, heart, liver, spleen, kidney, lung, intestine and muscle) were harvested and weighed. Subsequently, radioactivity uptake was determined in *γ*-counter. Activity concentrations in the tissues were calculated as percentage of the injected dose per gram of tissue (%ID/g). To correct for radioactive decay, injection standards were counted simultaneously.

### Whole-body micro single-photon emission computed tomography/computed tomography imaging

SPECT/CT scans and images were obtained with a Micro SPECT/CT PLUS system (Bioscan; Washington DC) equipped with a 0.74-mm nine-pinhole collimator: SPECT: 140 keV, 30 s/frame, 256 × 256 matrix, 20 % window; CT scanner: 55 kVp, exposed time: 1,000 ms, 180° plane. Static scans of 12 tumor-bearing mice (6 HEP-2, 6 CNE-1) were obtained 60 min after tail vein injection of approximately 37–55.5 MBq of ^99m^Tc-3P-RGD2 in 0.1 mL saline. All 12 mice were anesthetized with 1.5 % isoflurane for micro SPECT/CT and throughout imaging. It took 30 min to complete the whole-body SPECT scan and 15 min to complete the whole body CT scan. SPECT and CT data were reconstructed using InvivoScope software (Bioscan; Washington DC). The volumes of interest were drawn manually to cover the entire tumor. Based on the view in the CT image, the soft, non-tumor tissue reference (in the same trans-axial plane, muscle) was also marked, and the T/NT ratios were calculated.

### Immunohistochemical staining

For immunohistochemical investigation, formalin-fixed, paraffin-embedded tumor tissues from mice were sectioned (5 μm) and stained using the biotinylated monoclonal anti-*α*_v_ *β*_3_ antibody (1:100, rabbit IgG; Beijing Biosynthesis Biotechnology, China). The appropriate secondary antibody (Rabbit SP Kit, Beijing Biosynthesis Biotechnology, China) was used. Sections were processed by peroxidase staining (Diaminobenzidine Kit, Beijing Biosynthesis Biotechnology, China).

Light microscopic evaluation of the density of integrin *α*_v_ *β*_3_ was done according to the Fromowitz method [27]. Staining intensity was determined in five adjacent microscopic fields using a ×40 magnifying lens and a ×10 ocular. The staining positivity was graded on a four-point scale: 0 = no staining, 1 = weak, 2 = moderate, and 3 = strong positivity. The percentage of cells at each intensity was graded on a five-point scale: 0 = 0 %, 1 = 1–25 %, 2 = 26–49 %, 3 = 50–80 %, and 4 = >80 %. The overall staining intensity score was calculated as staining positivity score + percentage of cells score.

### Statistical analysis

All statistical analyses were done using SPSS 11.5 (SPSS Inc, USA). Data are presented as mean ± SD. The correlation between quantitative parameters was evaluated by linear regression analysis and calculation of Pearson’s correlation coefficient. Student *t*-tests for unpaired data were conducted to determine the significant differences between the groups in the studies of imaging and biodistribution. Statistical significance was defined as a *P* value < 0.05.

## Results

### Radiochemical purity of ^99m^Tc-3P-RGD2

**Fig. 1:**
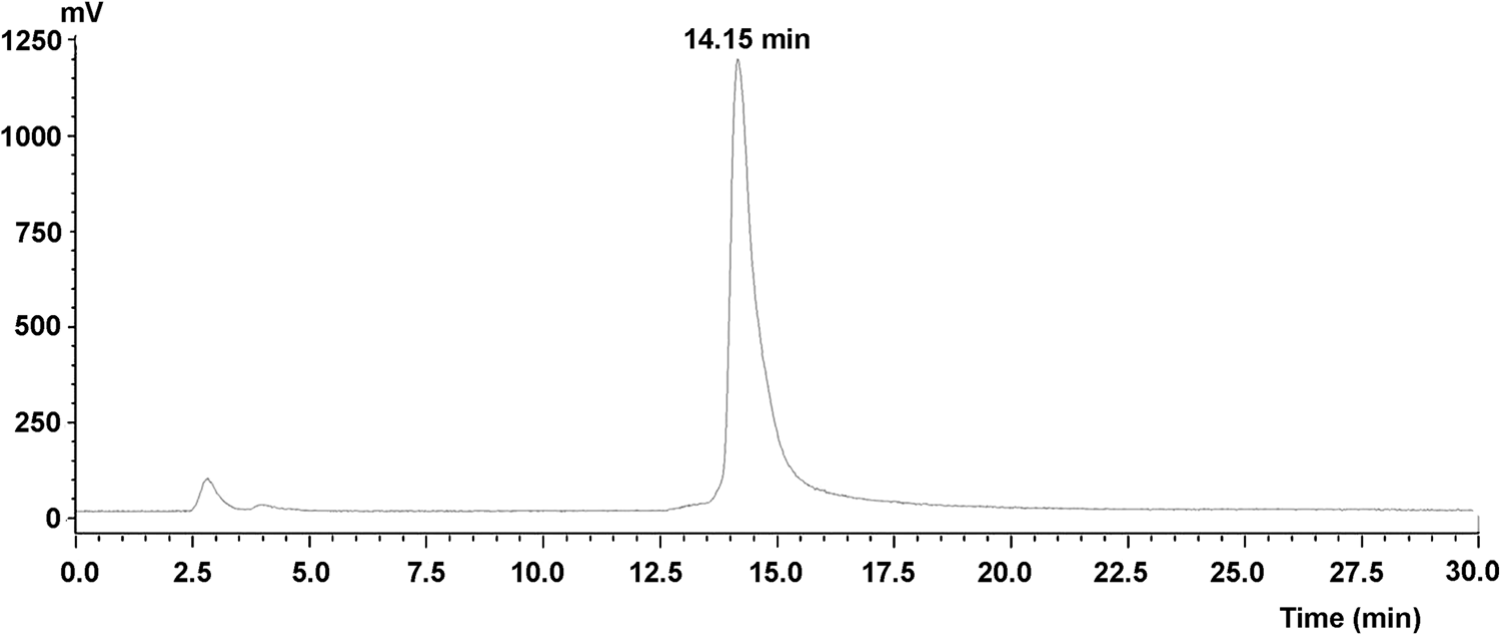
Radio-HPLC chromatograms for ^99m^Tc-3P-RGD2

The radiochemical purity of ^99m^Tc-3P-RGD2 was determined by radio-HPLC. As shown in Fig. 1, the retention time of ^99m^Tc-3P-RGD was 14.15 min and the radiochemical purity was over 95 % after preparation.

### In vitro stability of ^99m^Tc-3P-RGD2

**Fig. 2:**
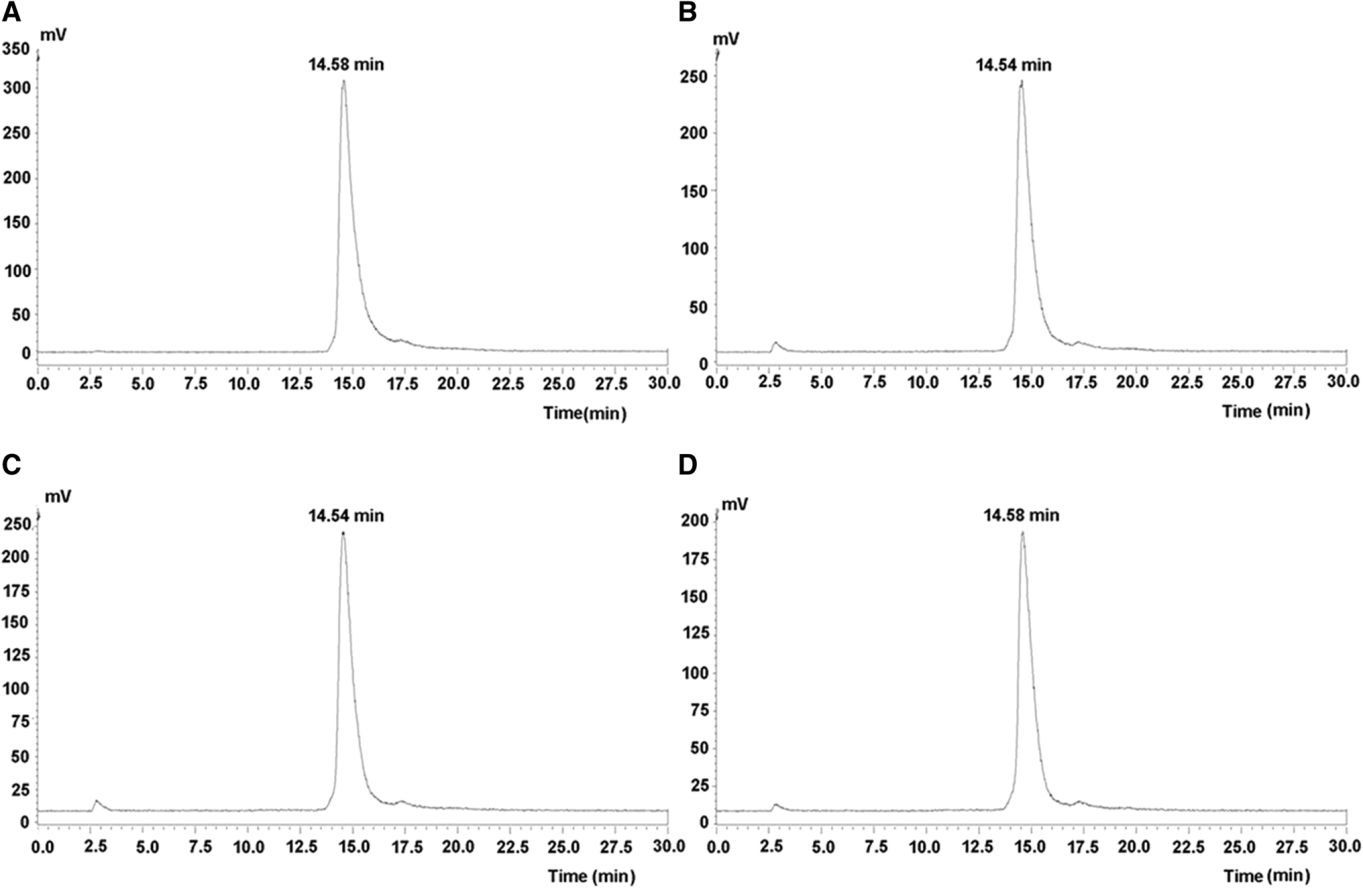
Radio-HPLC analysis of ^99m^Tc-3P-RGD2 stability. ^99m^Tc-3P-RGD_2  _incubated in new-born calf serum for 0 min (**a**), 3 h (**b**), 4 h (**c**), 6 h (**d**) after labeling

As shown in Fig. 2, HPLC analysis results for ^99m^Tc-3P-RGD2 indicated that it was stable in fetal calf serum after incubation for 6 h.

### Expression levels of integrin *α*_v_*β*_3_ in HEP-2 and CNE-1 cells

**Fig. 3:**
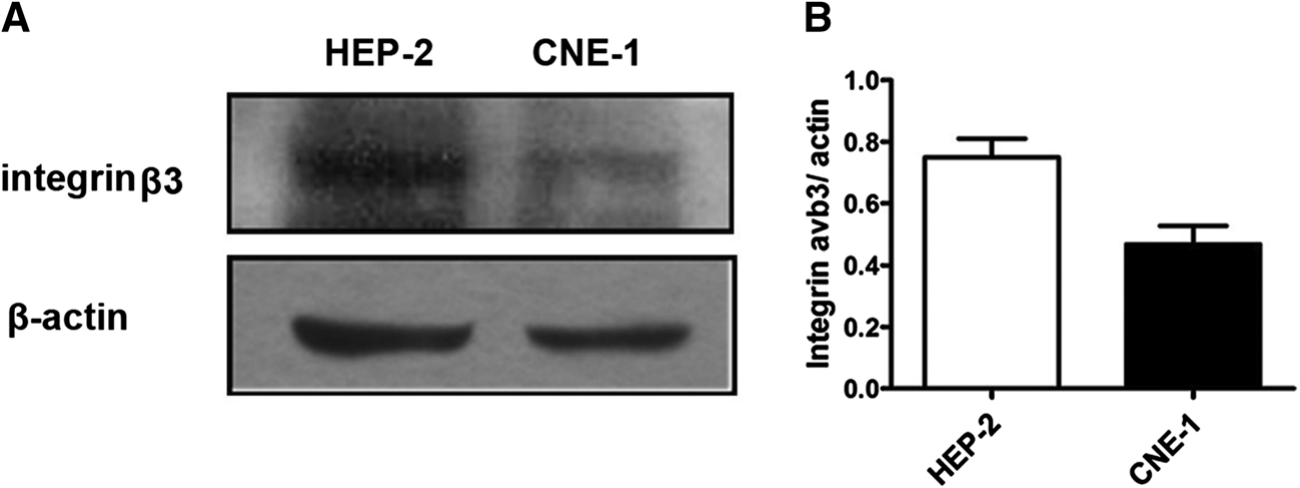
Integrin *α*_v_ *β*_3_ protein expression in HEP-2 and CNE-1 cells. **a** Western blot analysis for integrin*β*_3_ expression in HEP-2 and CNE-1 cells and (**b**) quantification for (**a**)

The expression level of integrin *α*_v_ *β*_3_ was detected in both cell lines. As shown in Fig. 3, integrin *α*_v_ *β*_3_ was expressed higher in HEP-2 cells than in CNE-1 cells.

### Biodistribution

**Fig. 4:**
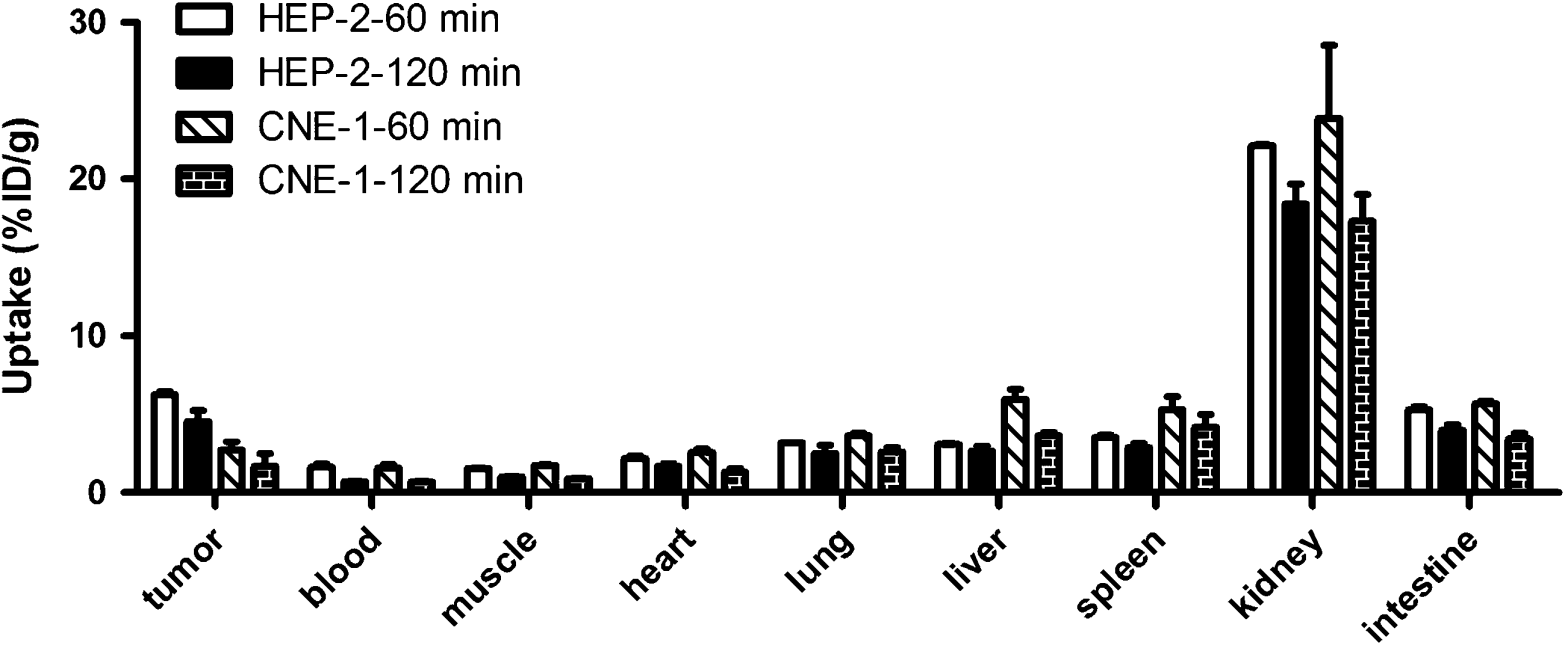
Biodistribution of ^99m^Tc-3P-RGD2 in HEP-2 and CNE-1 tumors at 60 or 120 min after tracer injection. Data are mean ± SD

The biodistribution of ^99m^Tc-3P-RGD2 in HEP-2 and CNE-1 tumor models were summarized as Fig. 4. In the HEP-2 model, the tumor uptake of ^99m^Tc-3P-RGD2 was moderately high (6.25 ± 0.22 %/g) at 60 min p.i., and its tumor washout was relatively high (4.56 ± 0.67 %/g at 120 min p.i.). In the CNE-1 model, the tumor uptake of ^99m^Tc-3P-RGD2 was relatively low (2.74 ± 0.51 %/g) at 60 min p.i., and its tumor washout was also high (1.69 ± 0.18 %/g at 120 min p.i.). The tumor uptake of ^99m^Tc-3P-RGD2 in the HEP-2 model was significantly higher than that in the CNE-1 model at 60 or 120 min p.i. (Student *t* test, *t* values = 10.92 and 7.17 respectively,all *P* < 0.05). The kidney uptake of ^99m^Tc-3P-RGD2 was highest in the selected organs, indicating the radiotracer was excreted predominantly via the renal route. The uptake of ^99m^Tc-3P-RGD2 in blood and muscle was low at 60 min p.i., and its blood or muscle clearance was relatively high. In the HEP-2 and CNE-1 models, its tumor/blood ratios were 6.37 ± 0.68 and 2.49 ± 0.09 respectively at 120 min p.i., and its tumor/muscle ratios were 4.44 ± 0.42 and 1.86 ± 0.07 respectively at 120 min p.i.. The lower distribution in blood and muscle and higher uptake in tumor guarantees ^99m^Tc-3P-RGD2 as a valuable tracer to monitor cancers.

### Uptakes of ^99m^Tc-3P-RGD2 in HEP-2 and CNE-1 xenografts

**Fig. 5:**
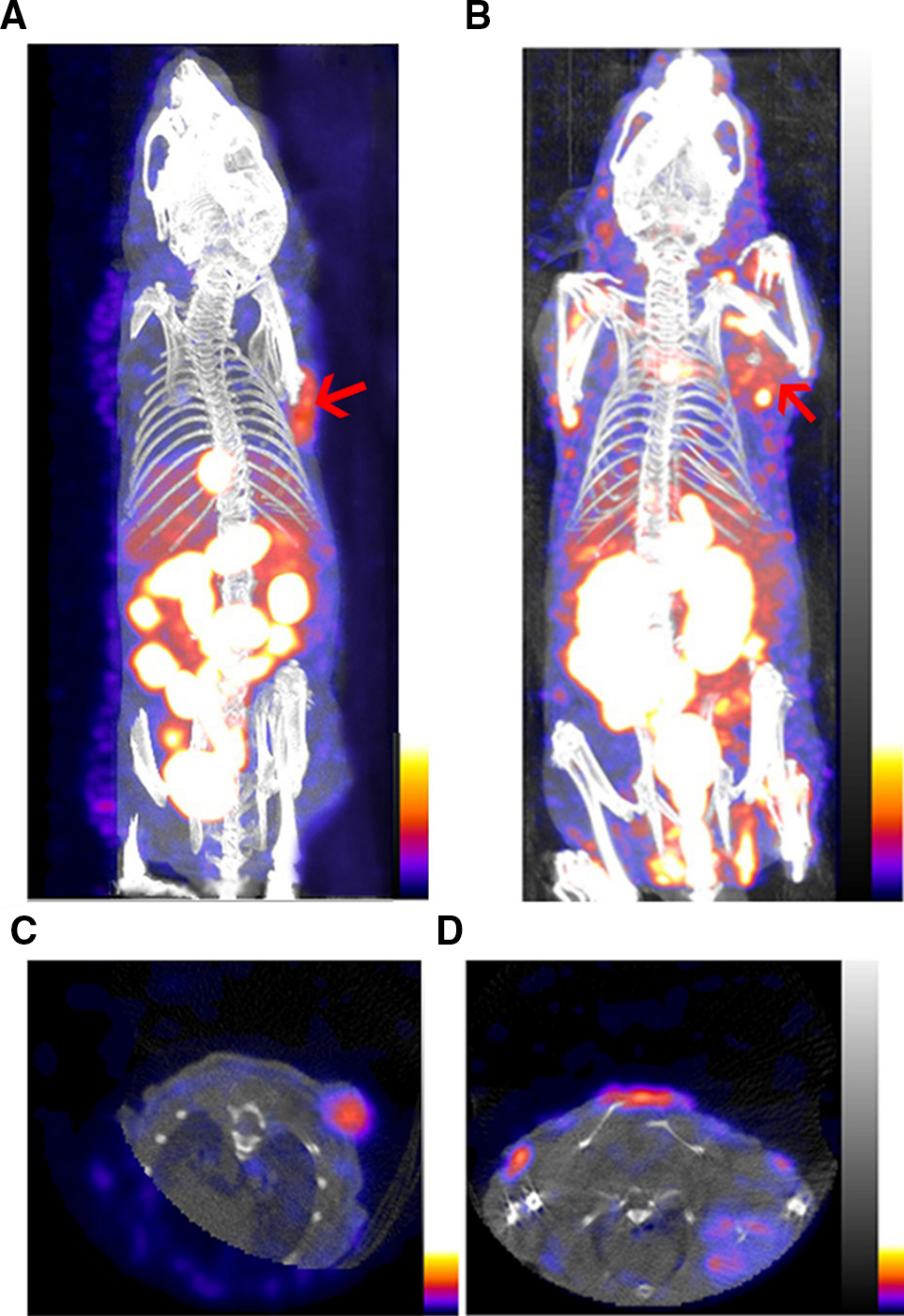
3D and transverse views of Micro SPECT/CT images of **a** and **c** nude mice bearing HEP-2 tumor and **b** and **d** nude mice bearing CNE-1 tumor 3 h after intravenous injection of ^99m^Tc-3P-RGD2

Representative whole body scans of HEP-2 and CNE-1 tumor-bearing mice at 3 h after intraperitoneal administration of ^99m^Tc-3P-RGD2 were shown in Fig. 5. In the HEP-2 tumor models, the tumors appeared clear with high contrast to the contralateral background at 2 h post-injection, and the average T/NT ratio at 3 h was 5.08 ± 0.04. By contrast, in the CNE-1 tumor models, the tumor could be visualized with moderate tumor-to-background contrast at 2 h post-injection with an average T/NT ratio at 3 h of 3.54 ± 0.10. The HEP-2 tumor uptake of ^99m^Tc-3P-RGD2 was significantly higher than that of CNE-1 tumors (*t* = 11.83, *P* < 0.05).

### Integrin *α*_v_*β*_3_ expression in HEP-2 and CNE-1 tumor tissues

**Fig. 6:**
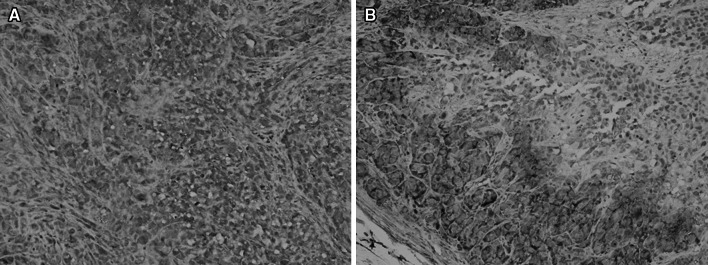
Immunohistochemical staning of the HEP-2 (**a**) and CNE-1 (**b**) tumor section using the anti-*α*_v_ *β*_3_ monoclonal antibody. The figures demonstrate intense staining predominantly of HEP-2 tumor tissue and circular peripheral staining of CNE-1 tumor tissue (×400)

Immunohistochemistry was used to examine the integrin *α*_v_ *β*_3_ expression levels in tumor tissues of different types of head and neck carcinoma, which was quantified by Fromowitz score. The Fromowitz score was 4.97 ± 0.37 in HEP-2 tumor and 2.60 ± 0.36 in CNE-1 tumor respectively. As shown in Fig. 6, integrin *α*_v_ *β*_3_ expression was higher in HEP-2 tumor tissues than in CNE-1 tumor tissues.

### Correlation of in vivo uptake of ^99m^Tc-3P-RGD2 and Integrin *α*_v_*β*_3_ expression

The uptakes of ^99m^Tc-3P-RGD2 were correlated well with the expression of integrin *α*_v_ *β*_3_ both in the HEP-2 model (Linear regression analysis, 120 min p.i.:* r* = 0.88,* P* < 0.05; 120 min p.i.: *r* = 0.97, *P* < 0.05), and CNE-1 model (Linear regression analysis, 60 min p.i.: *r* = 0.95,* P* < 0.05; 120 min p.i.:* r* = 0.97,* P* < 0.05).

## Discussion

Integrin *α*_v_ *β*_3_ is significantly up-regulated on activated endothelial cells and expresses a lot on various malignant tumor cells, but keep silent on resting endothelial cells or most normal organs [28–32]. Integrin *α*_v_ *β*_3_-targeted RGD provided a molecular imaging way to select high *α*_v_ *β*_3_ express patients and monitor therapy effect of patients receiving anti-angiogenic drugs or *α*_v_ *β*_3_ antagonists.

Radiolabeled RGD tracer should have high affinity and specificity for targeting integrin *α*_v_ *β*_3_. Several strategies have been adopted to improve integin *α*_v_ *β*_3_ binding affinity and specificity of the radiolabeled RGD peptide, such as the use of a multimeric cyclic RGD peptide and insertion of PEG4 spacers in the RGD dimeric molecule [33–35]. ^99m^Tc-3P-RGD2 has shown faithful effects in detecting carcinoma foci and great advantage in pharmacokinetics. In this study, we evaluated the feasibility of ^99m^Tc-3P-RGD2 imaging in BALB/c nude mice with HEP-2 and CNE-1 carcinoma xenograft. The results showed that ^99m^Tc-3P-RGD2 was not only sensitive in detecting the HNSCC foci but highly accumulated in the tumor tissue which was well corresponded with integrin *α*_v_ *β*_3_ expression level.

Our study demonstrated that ^99m^Tc-3P-RGD2 was excreted predominantly via renal route and lessly evacuated from the hepatobiliary route. The high clearance by kidneys, liver and intestine may minimize the background. However, to detect metastases within or close to kidneys, liver or intestine with this tracer may be limited in clinical practice as reported by previous study that liver metastases could image as hypointense lesions because of high background of ^18^F-RGD imaging [36]. The uptake of ^99m^Tc-3P-RGD2 by lung was slightly higher when calculated as ID %/g. However, lung contains much air in vivo, thus, it is reasonable to assume that lung metastases can be visualized clearly with high contrast to normal lung tissue.

## Conclusions

Our study demonstrated that the uptake of ^99m^Tc-3P-RGD2 in HNSCC correlated well with integrin *α*_v_ *β*_3_ expression and ^99m^Tc-3P-RGD2 SPECT/CT could be used as a non-invasive and effective method for monitoring integrin *α*_v_ *β*_3_ expression in HNSCC for proper patients accept individual therapy.
